# Engagement of Older Adults Receiving Home Care Services and Their Caregivers in Health Decisions in Partnership With Clinical Teams: Protocol for a Multimethod Study to Prioritize and Culturally Adapt Decision Aids for Home Care

**DOI:** 10.2196/53150

**Published:** 2023-11-20

**Authors:** Sabrina Guay-Bélanger, Emmanuelle Aubin, Marie Cimon, Patrick Archambault, Virginie Blanchette, Anik Giguere, Amédé Gogovor, Michèle Morin, Ali Ben Charif, Nouha Ben Gaied, Julie Bickerstaff, Nancy Chénard, Julie Emond, Julie Gilbert, Isabelle Violet, France Légaré

**Affiliations:** 1 VITAM - Centre de recherche en santé durable Centre intégré universitaire de santé et de services sociaux de la Capitale-Nationale Quebec, QC Canada; 2 Department of Family Medicine and Emergency Medicine Faculty of Medicine Université Laval Quebec, QC Canada; 3 Centre de recherche intégrée pour un système apprenant en santé et services sociaux Centre intégré de santé et de services sociaux de Chaudière-Appalaches Lévis, QC Canada; 4 Department of Anesthesiology and Intensive Care Université Laval Quebec, QC Canada; 5 Department of Human Kinetics and Podiatric Medicine Université du Québec à Trois-Rivières Trois-Rivières, QC Canada; 6 Department of Medicine Faculty of Medicine Université Laval Quebec, QC Canada; 7 CubecXpert Quebec, QC Canada; 8 Fondation Berthiaume-Du Tremblay Montreal, QC Canada; 9 L’Appui pour les proches aidants Montreal, QC Canada; 10 Centre intégré de santé et de services sociaux de Chaudière-Appalaches Lévis, QC Canada; 11 Centre intégré universitaire de santé et de services sociaux de la Capitale-Nationale Quebec, QC Canada; 12 Ministère de la santé et des services sociaux du Québec Quebec, QC Canada

**Keywords:** shared decision making, patient-centered care, home care, older adults, caregivers, decision aids, scalability assessment, innovation scalability self-administered questionnaire, interprofessional, team based

## Abstract

**Background:**

Older adults (people aged 65 years and older) face many difficult decisions. Patient decision aids (PtDAs) can help them and their families make informed value-congruent decisions. Some PtDAs have been developed for the home care context, but little is known about scaling them for use with older adults in a different culture.

**Objective:**

This study aims to (1) assess the scalability of existing PtDAs for older adults in the home care context; (2) prioritize those that best match the decisional needs of older adults in home care; and (3) culturally adapt the prioritized PtDAs so they can be scaled successfully to the Quebec health care system.

**Methods:**

This multimethod study includes 3 phases. All phases will be overseen by a steering committee of older adults, caregivers, health professionals, decision makers, community organization representatives, and researchers with the needed expertise. In phase 1, we will use the Innovation Scalability Self-administered Questionnaire, a validated scalability self-assessment tool, to assess the scalability of 33 PtDAs previously identified in a systematic review. Based on their scalability, their quality (based on the International Patient Decision Aids Standards), and the importance of the decision point, we will retain approximately a third of these. In phase 2, we will conduct a 2-round web-based Delphi to prioritize the PtDAs selected in phase 1. Using a snowball recruitment strategy, we aim to recruit 60 Delphi participants in the province of Quebec, including older adults, caregivers, health professionals, decision makers involved in home care services, and PtDA experts. In the first round, we will ask participants to rate the importance of several PtDA decision points according to various criteria such as prevalence and difficulty on a 5-point Likert scale (1=not important to 5=very important). Approximately 6 of the highest-rated PtDAs will be retained for presentation in the second round, and we will select up to 3 PtDAs judged as having the highest priority for cultural adaptation. In phase 3, using the Chenel framework and user-centered design methods, we will update and adapt the PtDAs to the Quebec health care system and integrate these PtDAs into an interprofessional shared decision-making training program for home care teams. The adapted PtDAs will respect the International Patient Decision Aids Standards criteria.

**Results:**

This study was funded in March 2022 by the Canadian Institutes of Health Research. Data collection for the web-based Delphi began in October 2023. Results are expected to be published in May 2024.

**Conclusions:**

This project will provide relevant and culturally appropriate decision support tools for older adults making difficult decisions and their home care teams that will be ready for scaling across the province of Quebec.

**International Registered Report Identifier (IRRID):**

PRR1-10.2196/53150

## Introduction

In Canada, the number of people aged 65 years or older has tripled over the past 40 years, and the current population is expected to grow by 68% by 2037 [1]. In the province of Quebec, Canada, people aged 65 years or older represent 21% of the population and should reach 26% by 2041 [2,3]. While 6.5% of all adults have received home care services in Quebec, this proportion rises to 28% among adults aged 75 years or older [4]. Thus, the demand for health and social services for older adults within the context of home care is likely to increase in the coming years. As older adults lose their autonomy and face increasing health problems, health decisions become more frequent as well as more difficult and decisions faced within the home care context present specific challenges [5].

Shared decision-making (SDM) is a collaborative process whereby clinical teams support individuals and their families to make informed health and social care decisions based on the best evidence and what matters most to them [6,7]. It fosters informed consent [8] and improves care experiences [9,10], health outcomes [10-12], clinical team well-being, and efficiency [13,14]. SDM also reduces the uptake of unproven treatments and, consequently, reduces health care waste [15]. It supports health equity, since the most vulnerable may benefit the most [16-18]. Finally, SDM has been adapted to and successfully tested in the home care context among interprofessional teams [19-21]. It was shown that home care interprofessional teams trained in SDM, along with older adults and their caregivers who had benefited from a decision aid, were more likely to share decisions together about housing transitions [19,20,22-24].

Patient decision aids (PtDAs) are tools that support people in defining the decision to be made, provide information regarding options and outcomes, and help clarify personal values and preferences [25]. They have been successfully used to bolster SDM [26] and to promote the involvement of family members in health decisions [19]. They have been shown to increase participants’ knowledge, decrease their decisional conflict, and reduce the number of people who were passive or undecided in decision-making. They also improve patient‐clinician communication and patient satisfaction with their decisions [16,18,27].

However, the uptake of SDM and PtDAs in Canada has been disappointing [15]. In a 2018 survey of 1591 Canadians, only 36% of older adults reported that they were always or often offered choices, and only 35% were always or often asked what was important to them. Among them, 8.9% reported receiving home care services, and results showed that they were the least engaged in decisions about their health [28]. In order to address this inequity in decision support, we conducted 2 pan-Canadian surveys and found that older adults in the home care context face numerous difficult decisions that fall under 3 main areas: housing and safety, managing health conditions, and end-of-life care [5,29]. In another survey, health care professionals reported that older adults and their family members needed more help in obtaining information about available options, dealing with peer pressure, and navigating the health care system [30].

We hypothesize that widespread uptake of PtDAs will be more likely if they are adapted to the context in which they will be used [31-33]. Most PtDAs are created to target 1 decision point, 1 defined population, and 1 context or health care system, but when they are scaled to further contexts, they rarely address the specificities of the stakeholders or health care systems into which they will be integrated. Rather than creating a new PtDA for each context, the adaptation of existing PtDAs has numerous benefits: it reduces the duplication of development efforts, optimizes the use of resources, improves relevance and applicability, and engages knowledge users in target locations [34].

Therefore, we propose the ENGAGE (Engagement of Older Adults Receiving Home Care Services and Their Caregivers in Health Decisions in Partnership With Clinical Teams) study: an integrated knowledge mobilization (iKM) scaling project whose aim is to assess the scalability of existing home care PtDAs, prioritize them in relation to the decisional needs of older adults in home care, and culturally adapt them so they can be scaled successfully to the Quebec health care system.

## Methods

### Overview of Study Design and Context

The ENGAGE study is a multimethod study with 3 phases: (1) a scalability assessment, (2) the prioritization of existing PtDAs, and (3) cultural adaptation of the most relevant PtDAs to the specificities of the stakeholders and the Quebec health care system. Each phase will use a distinct methodology to meet the objectives.

The ENGAGE study will be conducted within the Quebec health and social care system, 1 of 14 independent health and social care systems in Canada. Each of these systems (1 in each province or territory and 1 at the federal level) is responsible for coordinating, insuring, and managing care and services for the population. Within Quebec, health care services, including home care services, are provided by 18 regional authorities [15,35]. Since the province of Quebec has its own health care system and regulations, and its population is mostly French speaking, PtDAs need careful and culturally sensitive translation and need to be adapted to the Quebec situation, for example, include options available specifically in Quebec, to ensure their uptake by the population [36].

Throughout the project, we will use an iKM approach to strengthen our partnerships with knowledge users and other interested parties [37]. We will form a steering committee of 8-10 persons to oversee the operationalization of the project. The committee will involve older adults, caregivers, health professionals, decision makers in health organizations offering home care services, representatives of community organizations offering services to older adults and caregivers, and representatives of the provincial health ministry. The committee will also include researchers with the expertise needed to conduct the project, that is, experts in PtDAs, the interprofessional SDM approach, Delphi design, scaling, and research with older adults receiving home care services. We will recruit the committee through our networks in 2 regional health authorities (1 rural and 1 urban). We will organize at least 6 meetings over the course of the project to ensure the engagement of committee members in all phases of the project. We will also send quarterly newsletters to all knowledge users and partners to inform them of the project’s progress. We will remain attentive to the needs of the knowledge users and will respect the constraints of the managers and workers in the clinical settings as well as the older adults and their families. We will also contribute to capacity building by including trainees in all phases of this project. Finally, considerations of sex, gender, equity, diversity, and inclusion will be considered at all stages of the project using the Canadian Institutes of Health Research (CIHR) guidelines [38,39].

To ensure accurate reporting of the iKM approach, we will report results using the Guidance for Reporting Involvement of Patients and the Public (GRIPP2) checklist [40]. We will also report on the adaptation of PtDAs according to the Guidance for Reporting Intervention Development Studies in Health Research (GUIDED) checklist [41].

### Phase 1: Scalability Assessment of the Existing PtDAs

We recently completed a systematic review of PtDAs developed for home care [42]. For them to reach the largest proportion of people who can benefit from them, PtDAs for home care use must be analyzed for their scalability. Scalability is the ability of a health innovation that has demonstrated effectiveness under controlled conditions to be expanded in real-life circumstances to reach a larger proportion of the eligible population while still maintaining effectiveness [43]. Our team developed and tested the Innovation Scalability Self-administered Questionnaire (ISSaQ), a validated evaluation tool for assessing the scaling potential of health innovations that is designed to be used by teams planning to scale health and social services innovations [44,45]. ISSaQ is a list of 37 items covering 12 essential components of scalability, such as evidence on the effectiveness of the innovation, its characteristics, its acceptability, its adaptability, and the quantifiable costs and benefits of scaling (Multimedia Appendix 1, in French only). We will therefore use the ISSaQ to assess the scalability of each of the 33 PtDAs developed for home care that were identified in our systematic review [42].

First, we will retrieve all available information on each of the PtDAs included in our systematic review by searching for publications. A total of 2 team members will then independently extract the characteristics of the PtDAs (eg, type, purpose, decision point, target population) and complete the ISSaQ scalability assessment grid for all 33 PtDAs identified in the literature [42]. Any disagreements will be discussed with a third team member to reach a consensus. The results will be synthesized and discussed with the steering committee. Given the large number of PtDAs identified in the literature review (n=33), the steering committee will then select approximately one-third of the PtDAs based on the relevance of the decision point to home care for older adults in the experience of the steering committee, their scalability, and their quality according to the international criteria established by the International Patient Decision Aid Standards (IPDAS) Collaboration [46]. In order to reach a consensus on the final selection, steering committee members will vote according to all 3 criteria mentioned above, and PtDAs receiving the highest number of votes will be selected for the next phase.

### Phase 2: Prioritization of PtDAs Selected in Phase 1

#### Study Design

We will conduct a web-based Delphi (e-Delphi) study to prioritize the PtDAs selected in phase 1 [47]. The RAND research organization at the University of California at Los Angeles (RAND/UCLA) appropriateness method will be used to judge the prioritization and relevance of the selected PtDAs (maximum of 10 PtDAs) [48]. The decision point criteria deemed necessary for prioritization will be determined by the steering committee. These criteria could include, for example, the prevalence of the decision addressed by the PtDAs, the level of difficulty of the decision, the date at which the evidence was updated, and its potential for scale-up. A questionnaire will then be developed that addresses the various decision point criteria identified as priorities.

#### Delphi Participants

##### Recruitment

We aim to recruit 60 participants in the province of Quebec for this phase of the study to ensure a minimum of 40 participants in both rounds of the e-Delphi study [49,50]. We will use a snowball recruitment strategy, a nonprobability sampling method whereby study participants are asked to recruit future participants from among their social networks [51]. We will recruit older adults, caregivers, health professionals, decision makers involved in home care services (eg, home care team managers, directors of community organizations, managers of health care organizations, and government deputy health ministers or advisers), researchers, and PtDA experts. We aim to recruit a minimum of 8 to 12 participants for each of these categories to ensure representativeness [49]. Inclusion criteria for older adults will be as follows: (1) aged 65 years or older; (2) has home care experience; (3) can participate in the specified timeframe; (4) can read and understand French; and (5) can give informed consent. Inclusion criteria for other categories of participants will be the same except for the following: (1) aged 18 years or older and (2) has home care experience as a caregiver, health professional, decision maker, or expert. We plan to recruit participants using the snowball method with the help of our steering committee members, partners, and knowledge users.

##### Older Adults and Caregivers

We will ask our partners about the possibility of recruiting potential candidates from their organizations. Our partners in relevant organizations will be invited to share the invitation email with their members and through their social media platforms. The clinical teams in home care working for 2 regional health and social service authorities (1 rural and 1 urban) will also be invited to share the invitation with the older adults and caregivers they meet during their home care appointments. If necessary, with the agreement of the potential participants, the names and telephone numbers of interested individuals may be shared with the research team so that a team member can contact them, explain the project further, and invite them to participate.

##### Health Care Professionals

Health care professionals will be recruited with the help of managers from the 2 regional health and social services authorities. We will ask them to distribute an invitation email to the health professionals in their organizations. If necessary, we will contact home care managers in other health authorities in the province of Quebec.

##### Decision Makers

We will solicit our partners who are already in decision-making roles to disseminate the invitation within their networks and help us identify other decision makers.

##### Other Experts

Finally, members of our research team and collaborating teams will circulate the invitation by email to their respective networks to help us identify potential PtDA experts who can manifest their interest by contacting the team members responsible for the study.

We will make sure all participants understand our research objectives as we prepare and oversee the information provided to them. With a concern for equity, we will also solicit older adults and family members in rural areas, whom we will reach in person if necessary.

#### Data Collection

The e-Delphi consensus study will be conducted over the web using the REDCap (Research Electronic Data Capture; Vanderbilt University) tools hosted at Université Laval [52,53]. Participants will read and sign a consent form in the REDCap platform before completing the questionnaire. We anticipate that 2 rounds will allow for an acceptable degree of agreement on the PtDAs to be prioritized. There will be an interval of approximately 3 weeks between the 2 questionnaires. Participants who do not have easy access to a computer will be invited to complete the questionnaire during an in-person meeting with a research assistant. For each round, participants will be asked to submit their completed questionnaire within 2 weeks. An email reminder will be sent 48 hours before the end of the 2-week period. In the first round, we will collect participants’ sociodemographic information such as age, sex, gender, and education level. For each of the PtDAs selected in phase 1 (maximum of 10 PtDAs), we will present the main characteristics of the PtDAs (eg, decision point addressed, year, and country of development) and an image of each PtDA. Then we will ask participants to rate the PtDA according to the decision point criteria prioritized by the steering committee (eg, prevalence of the decision, level of difficulty of the decision, and potential for scale-up) included in the questionnaire on a 5-point Likert scale (1=not important to 5=very important). Participants will also be able to add free-text comments. We will calculate the overall scores and summarize free-text comments. Results will be synthesized and discussed with the steering committee. The highest-rated PtDAs will be retained for the next round (approximately 6 PtDAs).

In the second round, we will repeat the same procedure, but only the PtDAs prioritized in the first round will be presented (approximately 6 PtDAs). The results of the second round will be summarized and they will be presented and discussed with the steering committee. We will then select up to 3 PtDAs judged as having the highest priority for cultural adaptation (phase 3).

#### Data Analysis

Descriptive analyses of sociodemographic data will be conducted to profile the participants. Descriptive analyses will also be used to calculate the overall scores for each of the PtDAs after the 2 e-Delphi rounds. Regarding relevance and clarity, if the number of participants giving a score of 4 to 5 for each dimension divided by the total number of participants is greater than 0.80, this will be considered a good score. Regarding necessity, the formula (*Ne* – *N* / 2) / (*N* / 2) will be applied, where *Ne* is the number of participants indicating an element as important and *N* is the total number of participants. This indicator varies between –1 and +1, with higher scores indicating consensus. Results will be collated using SAS (version 9.0; SAS Institute Inc) to calculate the median score and percentage of agreement. We will summarize the open-ended comments using thematic analysis.

### Phase 3: Adaptation of Prioritized PtDAs to the Specificities of the Stakeholders and Health Care Systems Into Which They Will Be Integrated

Chenel et al [54] propose a framework for cultural adaptation of PtDAs in 4 stages: exploration (ie, identification of content to be adapted), adaptation (ie, incorporation of modifications), pilot testing (ie, validation with knowledge users), and field testing (ie, validation in a real-world context). Informed by this framework, we propose to pilot the cultural adaptation of the selected PtDAs using user-centered design methods and to set up a process that will serve as a proof of concept for the adaptation of the PtDAs to Quebec.

Specifically, with the help of the steering committee and the extended knowledge user group involved in this project, we will translate the selected PtDAs into French and identify the content that needs to be adapted, such as evidence updates and local information on available options. The research team will then incorporate these changes into the new versions of the selected PtDAs. We will engage language editors or graphic design services as needed. We will then validate the new versions with the steering committee members and our extended knowledge user team, using a new collaborative web-based platform for designing printable and interactive PtDAs [55]. This user-tested platform (PADA) was developed by an international team and enables the collaborative design, revision, or adaptation of PtDAs for multiple needs and contexts. PADA also facilitates the collection and integration of knowledge users’ feedback [56]. Agendas and minutes of each meeting will be produced to document the process of adapting the PtDAs to the Quebec cultural context. The final step will be to integrate the adapted PtDAs into an interprofessional SDM training program. Our team produced and contributed to the first interprofessional model of an SDM training program [57,58] and developed with knowledge users an interprofessional SDM training program for the use of a PtDA for housing transitions that was well received in home care settings [19-21,59]. We will develop a new generic version of this interprofessional SDM training program that can be used in conjunction with the selected PtDAs or any other PtDA.

### Ethical Considerations

This project was approved by the ethics committee of the Centre intégré universitaire de santé et de services sociaux de la Capitale-Nationale (MP-13-2023-2848). All stages of this research project will be carried out in accordance with Canadian procedures for informed consent (Tri-Council Policy Statement: Ethical Conduct for Research Involving Humans) [60]. These procedures include disclosure of benefits, risks, and drawbacks of participation, documentation of consent, patient or surrogate competency, plain language, the voluntary nature of the decision, consent for future use of data, confidentiality of data, and data management practices. All participants will give informed consent before participating in the study.

## Results

This study was funded in March 2022 by the CIHR. This protocol was initially submitted before any participants were recruited. The steering committee was recruited in June 2023 and is ready to begin its work. The committee consists of 2 older adults or caregivers; 1 representative of an organization whose mission is to improve services for older adults; 2 health professionals who are also decision makers in 2 health organizations offering home care services in the province of Quebec; 1 representative of the provincial health ministry; 1 geriatric internist with expertise in research with older adults receiving home care services; 1 family physician with expertise in SDM, PtDAs, scalability assessment, and e-Delphi studies; and 1 junior researcher with expertise with SDM and PtDAs. The consensus meeting with the steering committee to select the PtDAs to be prioritized in the e-Delphi study was held in September 2023. Data collection for the e-Delphi study began in October 2023. Results are expected to be published in May 2024.

## Discussion

This study will provide information about the scalability of PtDAs relevant to home care, a prioritized list of PtDAs for home care, and up to 3 PtDAs culturally adapted to the specificities of the Quebec health care system and stakeholders in the home care context. The study will provide a proof of concept of the process of adapting the PtDAs and integrating them into an interprofessional SDM training program for the home care sector so they can be scaled successfully in the Quebec health care system.

This study has several notable strengths. First, this project was developed as part of a collaboration with knowledge users that dates back to 2007, and its objectives were developed in response to their stated needs [19,20,28,59,61-63]. We have assembled an experienced interdisciplinary team with a long history of collaboration who possess expertise in developing and adapting PtDAs for the home care sector. In addition, the goals of this project are supported by earlier studies conducted by our team since 2007 showing that older adults have unmet needs for support in making shared informed decisions [5,19,20,28-30,59,61-63] and that the engagement of older adults and their families in health and social care decisions is encouraged by the use of PtDAs and training of clinical teams in SDM [19,20,64,65]. Since most PtDAs are in English and were developed in high-income countries, another strength of this study is that it will contribute to the literature on the cultural adaptation of PtDAs, not only for the Canadian context but for any context where languages and cultures differ within the same region or country.

The main limitation is that the PtDAs we will prioritize have been identified in a systematic review of published literature [42]. It is therefore possible that these PtDAs do not represent all the current or emerging difficult decisions faced by older adults, their families, and stakeholders. During the COVID-19 pandemic, different decisional needs emerged among older adults that affected their care, such as “should I delay my surgery?” [66,67]. To address this limitation, if participants express a desire for new PtDAs that do not yet exist, we will use the generic Ottawa decision aid to create one that is culturally adapted to the Quebec context [68]. Finally, operational issues could include recruiting enough people for the e-Delphi study. If we are unsuccessful with our current methodology, we will ask the 32 home care teams who participated in our previous home care randomized trials to suggest additional potential members [19,21].

The ENGAGE project is a response to a declared urgent need by the Quebec government to address gaps in home care [69-72], including the need to support and empower older adults and their families in health-related decision-making. This project will provide relevant and culturally appropriate decision support tools for older adults making difficult decisions and their home care teams. The resulting tools will be ready for scaling across the province of Quebec.

## Appendix

Multimedia Appendix 1 Innovation Scalability Self-administered Questionnaire (ISSaQ).

Multimedia Appendix 2 Peer-review report by the Canadian Institutes of Health Research (CIHR/IRSC, Canada).

## Abbreviations

CIHR: Canadian Institutes of Health Research
ENGAGE: Engagement of Older Adults Receiving Home Care Services and Their Caregivers in Health Decisions in Partnership With Clinical Teams
GRIPP2: Guidance for Reporting Involvement of Patients and the Public
GUIDED: Guidance for Reporting Intervention Development Studies in Health Research
iKM: integrated knowledge mobilization
IPDAS: International Patient Decision Aid Standards
ISSaQ: Innovation Scalability Self-administered Questionnaire
RAND/UCLA: RAND research organization at the University of California at Los Angeles
REDCap: Research Electronic Data Capture
PtDA: patient decision aid
SDM: shared decision-making

## Data Availability

The data sets generated or analyzed during this study are available from the corresponding author on reasonable request.
