# Supplementary material for: Engagement of Older Adults Receiving Home Care Services and Their Caregivers in Health Decisions in Partnership With Clinical Teams: Protocol for a Multimethod Study to Prioritize and Culturally Adapt Decision Aids for Home Care
Source: JMIR Res Protoc. 2023 Nov 20;12:e53150. doi: 10.2196/53150 (PMC10696497; doi:10.2196/53150)
Supplement: Multimedia Appendix 1 [file resprot_v12i1e53150_app1.pdf]

UNITÉ DE SOUTIEN SSA | QUÉBEC
Ensemble pour un système de santé qui apprend

Questionnaire auto-administré sur le potentiel de mise à l'échelle des innovations en santé et en services sociaux– ISSaQ 4.0

Outil et guide d'utilisation

Roberta D.C. Corôa, Ali Ben Charif, Karine V. Plourde, Amédé Gogovor, France Légaré

(Mars 2023)

Auteur correspondant :

France Légaré

PARTENAIRES PRINCIPAUX
Instituts de recherche en santé du Canada
Ministère de la Santé et des Services sociaux
Fonds de recherche du Québec – Santé
RUISSS Université McGill
RUISSS Université de Montréal
RUISSS Université de Sherbrooke
RUISSS Université Laval

Université Laval
Université McGill
Université de Montréal
Université de Sherbrooke

Stratégie de recherche axée sur le patient
SRAP
Le patient d'abord

IRSC CIHR
Instituts de recherche en santé du Canada
Canadian Institutes of Health Research

Citation :

Corôa RDC, Ben Charif A, Plourde KV, Gogovor A, Légaré F. Questionnaire auto-administré sur le potentiel de mise à l’échelle des innovations en santé et en services sociaux– ISSaQ 4.0. Québec: Unité de Soutien SSA Québec; 2023 Mar p. 1–28.

Le présent outil est le résultat du projet « Développement d'un outil d'évaluation du potentiel de mise à l'échelle des innovations en soins de santé communautaires de première ligne (outil validé par un Delphi) », financé par l'Unité de Soutien SSA Québec dans le cadre des projets de transition 2021-2022. Le projet a été mené en collaboration avec la Chaire de recherche du Canada sur la décision partagée et l'application des connaissances, de l'Université Laval, et a reçu le soutien du Centre de recherche en santé durable – VITAM.

Les auteures et auteurs du présent document sont Roberta D.C. Corôa, Ali Ben Charif, Karine V. Plourde, Amédé Gogovor et France Légaré.

Nous remercions les chercheuses et chercheurs, patientes partenaires et autres collaboratrices et collaborateurs qui ont participé aux différentes étapes de ce projet :

Carole Thiébaut
Claude Bernard-Uwizeye
Diogo G.V. Mochcovitch
Georgina Suelene Dofara
Hervé Tchala Vignon Zomahoun
Jean-Sébastien Renaud
Kathy Kastner
Laura Ghiron
Léonel Philibert
Louisa Blair
Odilon Quentin Assan
Robert McLean
Samira Amil
Souleymane Gadio
Virginie Blanchette

Table des matières

1 Introduction....5

1.1 La mise à l'échelle des innovations en santé et en services sociaux....5

1.2 Le potentiel de mise à l'échelle....5

1.3 ISSaQ 4.0 : un outil basé sur les données probantes....6

2 Comment utiliser l'outil....7

2.1 Navigation....7

2.2 L'utilisation des échelles....7

2.3 Le besoin des données probantes....7

2.4 Documentation....8

2.5 Quand utiliser....8

2.6 À qui s'adresse l'outil....9

3. Glossaire.... 10

4. Fiche de l'innovation.... 12

5. Composantes du potentiel de mise à l'échelle (C).... 13

5.1 C1 : Problème social ou de santé adressé par la mise à l'échelle.... 13

5.2 C2 : Développement de la mise à l'échelle.... 14

5.3 C3 : Caractéristiques de l'innovation mise à l'échelle.... 15

5.4 C4 : Contexte politique de la mise à l'échelle.... 16

5.5 C5 : Efficacité de l'innovation mise à l'échelle.... 17

5.6 C6 : Coûts de la mise à l'échelle.... 18

5.7 C7 : Adaptabilité de l'innovation mise à l'échelle.... 19

5.8 C8 : Couverture de la mise à l'échelle.... 20

5.9 C9 : Acceptabilité de l'innovation mise à l'échelle.... 21

5.10 C10 : Milieu de mise à l'échelle.... 22

5.11 C 11 : Infrastructure requise pour la mise à l'échelle.... 23

5.12 C12 : Pérennisation.... 24

6. Ressources utilisées pour construire l'outil.... 25

1 Introduction

** La définition de tous les mots, termes et concepts marqués en bleu se trouve dans le glossaire à la page 11.*

1.1 La mise à l'échelle des innovations en santé et en services sociaux

La mise à l'échelle des innovations en santé et en services sociaux comprend des efforts pour accroître l'impact des innovations qui se sont montrées efficaces dans des contextes locaux ou expérimentaux, par la diffusion, la réplication ou l'adaptation d'innovations en santé[i]. Il s'agit d'une pratique basée sur des données probantes qui vise à améliorer la qualité des soins et le bien être d'une population ciblée[ii]. L'extension rapide des actions visant à accroître la couverture vaccinale contre le COVID-19 est un exemple réussi de mise à l'échelle. Au cours des dernières années, la littérature scientifique et plusieurs organisations ont souligné sa pertinence en considérant cette pratique comme essentielle pour réduire les gaspillages et les inégalités en santé[iii].

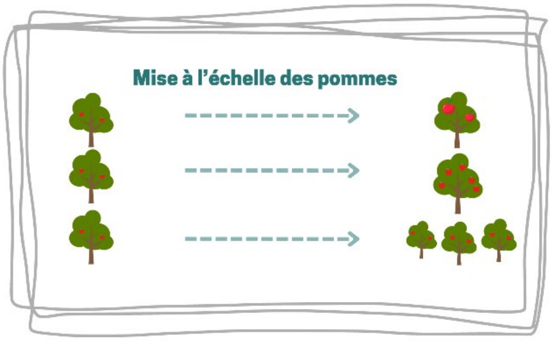

Adaptée de McLean et Gargani, 2022

1.2 Le potentiel de mise à l'échelle

L'évaluation du potentiel de mise à l'échelle d'une innovation est recommandée avant de procéder à la mise à l'échelle [iv]. Cette évaluation vise à assurer que l'innovation a le potentiel d'être diffusée, répliquée ou adaptée aux nouveaux contextes, tout en maintenant son efficacité et en augmentant par conséquent son impact[v].

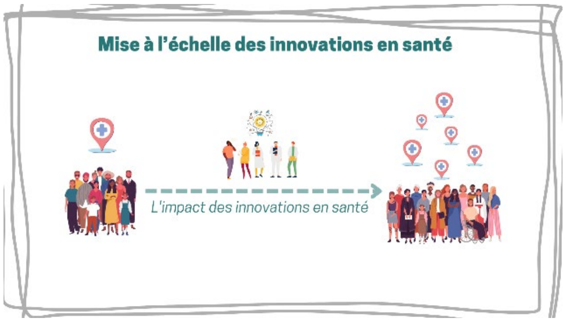

ISSaQ 4.0, 2023

1.3 ISSaQ 4.0 : un outil basé sur les données probantes

L'outil que vous avez en main permet aux équipes des innovations en santé et en services sociaux d'évaluer le potentiel de mise à l'échelle de leurs innovations. Il s'agit d'un questionnaire auto-administré contenant 37 énoncés distribués en 12 composantes du potentiel de mise à l'échelle[vi].

Composantes du potentiel de mise à l'échelle
1. Problème social ou de santé adressé par la mise à l'échelle
2. Développement de la mise à l'échelle
3. Caractéristiques de l'innovation mise à l'échelle
4. Contexte politique de la mise à l'échelle
5. Efficacité de l'innovation mise à l'échelle
6. Coûts de la mise à l'échelle
7. Adaptabilité de l'innovation mise à l'échelle
8. Couverture de la mise à l'échelle
9. Acceptabilité de l'innovation mise à l'échelle
10. Milieu de mise à l'échelle
11. Infrastructure requise pour la mise à l'échelle
12. Pérennisation de la mise à l'échelle

En utilisant cet outil, les innovatrices et innovateurs seront en mesure de prendre une décision éclairée sur la poursuite de la mise à l'échelle. En outre, l'outil leur permettra de visualiser les points faibles de leurs projets et de les améliorer pour une mise à l'échelle réussie.

La fiabilité de l'outil est fondée sur son processus de construction rigoureux, systématique et basé sur des données probantes. La **version 1.0** visait à évaluer le potentiel de mise à l'échelle des innovations en soins de première ligne et a été construite en 2017[vii], à partir d'une revue systématique[viii] et des guides reconnus de mise à l'échelle[ix]. La **version 2.0** apportait des petites modifications dans les libellés des questions, mais n'a jamais été publiée.

La **version 3.0** a été livrée à l'Unité SSA Québec en 2022. Pour élaborer cet outil, premièrement, une revue systématique a identifié dans la littérature les outils publiés portant sur l'évaluation du potentiel de la mise à l'échelle d'innovations en santé[x]. Cette étude a trouvé 21 outils, dont 320 items d'évaluation ont été extraits. Ensuite, 16 critères de sélection[xi] ont été appliqués sur ces items en vue d'assurer leur validité et pertinence. Dans l'approche d'application des connaissances intégrée (ACi), la liste des items sélectionnés a été révisée par des utilisateurs et des utilisatrices de connaissances externes à l'équipe de recherche du projet (y compris un chercheur, deux décisionnaires et une patiente partenaire). L'outil ISSaQ 3.0 contenait 45 énoncés distribués en 14 composantes du potentiel de mise à l'échelle. Sa portée exceptionnellement large a englobé les critères les plus pertinents pour évaluer le potentiel de mise à l'échelle des outils publiés à ce jour. En outre, cette version inclut des innovations en santé à tous les niveaux, la distinguant des versions 1.0 et 2.0.

La présente **version 4.0** est le résultat d'une étude Delphi qui a permis de valider et d'améliorer la version 3.0. Vingt-quatre (24) experts et expertes ont participé à l'étude, y compris des citoyen(ne)s et des patient(e)s, des décisionnaires, des membres des entités SRAP, des clinicien(ne)s, et des chercheurs(-euses). Ces participants ont évalué la pertinence, la clarté et la nécessité de chaque énoncé de l'outil et ont pu suggérer de nouveaux énoncés d'évaluation. Onze (11) énoncés n'ayant pas obtenu un score suffisant ont été exclus de l'outil et trois (3) nouveaux énoncés suggérés ont été inclus. Les questions et les commentaires reçus lors de l'étude Delphi nous ont permis d'améliorer ISSaQ en rendant les composantes et les énoncés de la version 4.0 plus clairs et plus précis.

2 Comment utiliser l'outil

2.1 Navigation

Chaque composante est introduite par une définition qui vous aidera à comprendre les énoncés présentés. Pour vous soutenir avec les termes et concepts utilisés, un glossaire est disponible à la fin de ce guide. Bien qu'il soit recommandé de suivre l'ordre proposé des composantes et des énoncés pour avoir une vue d'ensemble logique du processus de mise à l'échelle, il n'est pas obligatoire de le faire. Vous pouvez sauter certains thèmes si vous avez besoin de plus de temps pour réfléchir ou discuter avec l'équipe. Vous pouvez faire une pause et revenir à ISSaQ à tout moment pour continuer ou réviser votre évaluation. L'outil propose des espaces pour prendre des notes afin que vous puissiez enregistrer les informations que vous jugez importantes pendant son utilisation.

2.2 L'utilisation des échelles

L'outil propose des échelles de réponse où vous pouvez indiquer le chiffre correspondant à votre opinion sur les énoncés concernant la mise à l'échelle de votre innovation. Dans une échelle de 1 à 7, le 1 représente « Pas du tout d'accord » et le 7 « Tout à fait d'accord ». **Il est important de noter qu'il n'y a pas de score minimum ou idéal recommandé pour poursuivre la mise à l'échelle de votre innovation. Cependant, vos réponses devraient vous aider à réfléchir de façon éclairée sur le potentiel de mise à l'échelle de votre innovation avant de passer à l'étape suivante.**

2.3 Le besoin des données probantes

Plusieurs énoncés portent sur les données probantes disponibles sur l'innovation. Vous pouvez utiliser des données de différentes natures (quantitatives et/ou qualitatives), ainsi que des données publiées, déjà existantes ou produites spécifiquement sur votre innovation. Le plus important est qu'il s'agisse de données pertinentes et de qualité. La pertinence des données est

liée à la nature de l'innovation et à l'objectif de sa mise à l'échelle. Par exemple, des données qualitatives issues d'un petit échantillon d'entrevues pourraient être suffisantes pour appuyer la mise à l'échelle d'une innovation en santé visant à encourager les membres de la communauté à pratiquer la méditation, ce qui pourrait améliorer leur bien-être et leur santé mentale. Par contre, pour la mise à l'échelle d'un médicament, il est impératif de disposer de données robustes issues d'essais cliniques, avec des protocoles de contrôle des échantillons clairement définis, respectés et rapportés. Les critères d'évaluation de la qualité des données peuvent varier selon le type de données utilisées. Ils incluent des aspects tels que la méthodologie de collecte des données, l'échantillonnage, la validité des mesures et la fiabilité. Pour aider les équipes d'innovation à juger de la qualité de leurs données, nous les encourageons à établir des partenariats avec des équipes de recherche qualifiées.

2.4 Documentation

Il est recommandé de documenter toutes les données, réflexions et autres informations pertinentes survenant de l'utilisation de l'ISSaQ. Cette pratique facilitera la communication avec l'équipe et aidera à conserver une trace claire de l'évolution de mise à l'échelle de votre innovation. Vous pouvez créer un dossier pour chaque composante de l'outil pour classer les données ainsi que des notes de discussion et des documents supplémentaires. Cela vous permettra de réfléchir aux réponses fournies et de prendre vos décisions concernant la mise à l'échelle de façon éclairée. De plus, cette documentation vous aidera à justifier la mise à l'échelle à un niveau institutionnel plus large à l'avenir.

2.5 Quand utiliser

Vous pouvez utiliser ISSaQ lors des différentes étapes de la mise à l'échelle de l'innovation.

Avant l'implantation de l'innovation pilote : ISSaQ vous aidera à réfléchir sur le potentiel de mise à l'échelle dans le cadre de la recherche pour concevoir une innovation pilote ou lors de sa mise en œuvre. Cela vous permettra d'incorporer et de documenter les éléments qui seront nécessaires pour la mise à l'échelle de l'innovation dans de nouveaux contextes à l'avenir.

Avant la mise à l'échelle de l'innovation : Vous pouvez utiliser ISSaQ après la mise en œuvre d'une innovation pilote. L'évaluation du potentiel de mise à l'échelle est fortement recommandée lors de l'étape de préparation à la mise à échelle. Cela vous permettra d'identifier les forces et les faiblesses de la mise à l'échelle de l'innovation et facilitera la collecte des données et des informations nécessaires à la planification de ce processus.

Pendant ou après la mise à échelle de l'innovation : En cas d'utilisation rétroactive, l'outil permettra d'identifier les écarts et les éléments manquants ainsi que les raisons du succès de la mise à l'échelle de l'innovation, en contribuant à l'amélioration continue du processus. Cela est

recommandée dans le cadre des attentes de pérennisation et d’une plus grande institutionnalisation de la mise à l’échelle de l’innovation.

2.6 À qui s’adresse l’outil

L’outil ISSaQ a été conçu pour être utilisé pour toute personne ou organisation qui œuvre en santé dans les domaines de la recherche, des soins ou des services sociaux avec et pour les patientes et patients. Compte tenu de la complexité du processus de mise à l'échelle et de la spécificité de certains énoncés, nous recommandons que l'outil soit utilisé avec la participation de l'ensemble de l'équipe d'innovation. Cette équipe, à son tour, doit établir des collaborations avec toutes les parties prenantes de la mise à l’échelle, c’est-à-dire les personnes représentantes des utilisateurs et utilisatrices, des bénéficiaires, des organisations qui œuvre en santé dans les domaines de la recherche, des soins ou des services sociaux, des organismes subventionnaires et des décisionnaires qui doivent contribuer à partir de leurs perspectives au développement de la mise à l’échelle de l’innovation. Ainsi, l'organisation des données et des informations, ainsi que les réflexions, peuvent être partagées entre les membres du groupe, en fonction de leur expérience et de leurs possibilités.

Nous avons mobilisé les meilleures pratiques disponibles pour faire de la recherche inclusive et axée sur les patientes et patients. ISSaQ a été construit dans l’approche de l’application des connaissances intégrée et a reçu des contributions des patient(e)s et citoyen(ne)s partenaires dans toutes les étapes de sa construction. Ainsi, l’outil encourage l’engagement des patientes et patients et des citoyens et citoyennes à la mise à l’échelle. Toutefois, cet engagement nécessitera des efforts de la part des équipes d'innovation pour mener des discussions dans un langage accessible et pour bien cerner les compétences des patient(e)s et des citoyen(ne)s qui peuvent contribuer à l’évaluation du potentiel de mise à l’échelle de son innovation.

3. Glossaire

Composantes du potentiel de mise à l'échelle : Les composantes identifiées dans la littérature comme étant essentielles à la réussite de la mise à l'échelle d'une innovation en santé et en services sociaux.

Contextes locaux ou expérimentaux : Le contexte dans lequel une innovation a été mise en œuvre par la première fois.

Données pertinentes et de qualité : Les données pertinentes sont cohérentes, appropriées et répondent à une question spécifique ou à un objectif d'échelle. Les données de qualité sont fiables en termes de la méthodologie de collecte des données utilisée, de l'échantillonnage, et de la validité des mesures.

Données probantes : Les données obtenues par le biais de méthodes scientifiques rigoureuses, telles que des études expérimentales ou observationnelles, et peuvent inclure des données quantitatives (chiffres, statistiques) et/ou qualitatives (observations, témoignages).

Engagement des patientes et patients et des citoyens et citoyennes à la mise à l'échelle : Participation active des patientes et patients et des citoyens et citoyennes à la mise à l'échelle des innovations en santé et services sociaux à travers la collaboration et la coproduction des idées, plans, documents, outils, solutions et évaluations pendant toutes les étapes de ce processus.

Équipe de l'innovation : Équipe responsable pour la mise à l'échelle de l'innovation.

Groupes vulnérables : Personnes, communautés et groupes fréquemment confrontées à la discrimination et aux préjugés en raison de leurs caractéristiques ou positions dans la structure sociale. Ce sont les personnes d'ascendance africaine, les populations autochtones, les groupes Roms, Sinti et gens du voyage, les personnes appartenant à des minorités nationales ou ethniques, religieuses et linguistiques, les personnes migrantes, les réfugié(e)s, demandeurs(-euses) d'asile et personnes déplacées à l'intérieur de leur propre pays, les personnes vivant dans l'extrême pauvreté, les femmes, les personnes LGBTQI+, les personnes handicapées et les personnes âgées.

Infrastructure de suivi et d'évaluation : Les infrastructures, telles que l'équipement, les mesures et les ressources humaines qualifiées, pour accompagner et surveiller la mise à l'échelle d'une innovation.

Infrastructure organisationnelle : Les arrangements institutionnels nécessaires pour la mise à l'échelle d'une innovation, tels que les autorisations des directions, le comité de gouvernance et le soutien politique et communautaire.

Innovations en santé et services sociaux : Des nouvelles interventions, des interventions perçues comme nouvelles par leurs bénéficiaires ou des interventions adaptées à de nouveaux contextes.

Innovation pilote : L'innovation menée pour la première fois dans un contexte pilote ou expérimental.

Langage accessible : Mode de communication qui est compréhensible et utilisable par le plus grand nombre de personnes possible, y compris les personnes ayant des capacités ou des besoins différents.

Minorités visibles : Personnes, autres que les autochtones, qui ne sont pas de race blanche ou qui n'ont pas la peau blanche.

Mise à l'échelle : Des efforts pour accroître l'impact des innovations qui se sont montrées efficaces dans des contextes locaux ou expérimentaux, par la diffusion, la réplication ou l'adaptation d'innovations en santé.

Nouveaux contextes : Le contexte dans lequel l'innovation sera mise à l'échelle.

Obstacle politique : Des barrières au niveau de la prise de décision politique et de la mise en œuvre des politiques publiques qui peuvent constituer des obstacles pour la mise à l'échelle de l'innovation, tels que les conflits d'intérêts entre différents groupes, la bureaucratie, la résistance institutionnelle au changement et les contraintes légales et réglementaires.

Partenariats multipartites locaux : Les partenariats nécessaires à la mise à l'échelle de l'innovation provenant des différents secteurs, tels que les organismes financiers, les fournisseurs, les associations communautaires, les gouvernements, etc.

Parties prenantes de la mise à l'échelle : Les personnes représentantes des utilisateurs et utilisatrices, des bénéficiaires, des organisations et des personnes qui œuvrent en santé dans les domaines de la recherche, des soins ou des services sociaux, des organismes subventionnaires et des décisionnaires qui doivent contribuer à partir de leurs perspectives au développement de la mise à l'échelle de l'innovation.

Populations ciblées : La population et/ou les services qui bénéficieront de la mise à l'échelle de l'innovation.

Potentiel de mise à l'échelle : Potentiel d'une innovation d'être diffusée, répliquée ou adaptée aux nouveaux contextes tout en maintenant son efficacité et en augmentant par conséquent son impact.

Préparation à la mise à échelle : L'étape avant de la mise à l'échelle, y compris la mise en place de l'équipe, des partenariats, de l'infrastructure et de tout ce qui est nécessaire pour la poursuite. Quelques auteurs et auteures recommandent l'élaboration du plan de mise à l'échelle à cette étape.

Questions de sexe et genre : Aspects qui tiennent compte des inégalités sociales inhérentes aux relations de pouvoir dans notre société qui mettent en positions différentes les femmes et les hommes et les personnes cisgenre, transgenre et non binaires face à l'accès aux soins de santé.

Ressources humaines qualifiées : Personnel qualifié qui est soit déjà capable de travailler sur la mise à l'échelle de l'innovation, soit capable et disposé à recevoir une formation pour y parvenir.

4. Fiche de l'innovation

Nom de l'innovation :

Problème social ou de santé auquel s'adresse l'innovation :

Population ciblée par la mise à l'échelle :

Description de la mise à l'échelle :

Équipe de l'innovation et parties prenantes de la mise à l'échelle :

5. Composantes du potentiel de mise à l'échelle (C)

5.1 C1 : Problème social ou de santé adressé par la mise à l'échelle

Cette composante rassemble des énoncés liés au problème auquel s'adresse la mise à l'échelle de l'innovation. La mise à l'échelle doit répondre à un problème social ou de santé pertinent, ce qui signifie qu'elle doit bénéficier à une population ciblée et répondre à ses besoins en matière de santé et de bien-être. Il est donc important de prendre en compte les perspectives des parties prenantes et de la population ciblée pour décider sur la mise à l'échelle et de communiquer avec eux, en les faisant participer à toutes les étapes du processus.

Veuillez répondre à chacun des énoncés suivants en indiquant le chiffre qui décrit le mieux votre opinion :

	Pas du tout d'accord 1	2	3	4	5	6	Tout à fait d'accord 7
C1.1 Cette innovation* répond à un problème social ou de santé.	☐	☐	☐	☐	☐	☐	☐
C1.2 Il y a une demande explicite de mise à l'échelle de l'innovation par les parties prenantes*.	☐	☐	☐	☐	☐	☐	☐
C1.3 Il y a une demande explicite de mise à l'échelle de l'innovation par les populations ciblées*.	☐	☐	☐	☐	☐	☐	☐

* La définition des termes employés est présentée dans le glossaire.

Notes ✎

5.2 C2 : Développement de la mise à l'échelle

Cette composante rassemble des énoncés liés au processus de développement de la mise à l'échelle de l'innovation. Ce développement doit impliquer les parties prenantes et la population ciblée. Avoir un guide, un modèle ou un cadre théorique pourrait vous aider dans cette étape.

Veuillez répondre à chacun des énoncés suivants en indiquant le chiffre qui décrit le mieux votre opinion :

	Pas du tout d'accord 1	2	3	4	5	6	Tout à fait d'accord 7
C2.1 Les parties prenantes* ont donné leur perspective sur la mise à l'échelle.	☐	☐	☐	☐	☐	☐	☐
C2.2 Les populations ciblées* ont donné leur perspective sur la mise à l'échelle.	☐	☐	☐	☐	☐	☐	☐

* La définition des termes employés est présentée dans le glossaire.

Notes ✎

5.3 C3 : Caractéristiques de l'innovation mise à l'échelle

Cette composante rassemble des énoncés liés aux aspects éthiques et sociaux de l'innovation mise à l'échelle. Il s'agit d'assurer que la mise à l'échelle contribuera à accroître la justice sociale et non les inégalités sociales et en matière de santé. Les innovations doivent être conçues pour traiter avec respect et dignité les minorités visibles et les groupes vulnérables dans la société, tels que les femmes, les personnes LGBTQ2I, les populations racialisées, les personnes âgées et les personnes handicapées. Il est également important de garantir que l'innovation mise à l'échelle soit facile à comprendre pour les populations ciblées. En suivant ces principes, il est possible d'assurer que la mise à l'échelle bénéficiera à toute personne et contribuera à une société plus équitable et inclusive.

Veuillez répondre à chacun des énoncés suivants en indiquant le chiffre qui décrit le mieux votre opinion :

	Pas du tout d'accord 1	2	3	4	5	6	Tout à fait d'accord 7
C3.1 Les parties prenantes* impliquées partagent une vision commune de l'objectif de la mise à l'échelle.	☐	☐	☐	☐	☐	☐	☐
C3.2 L'innovation est facile à comprendre pour les populations ciblées*.	☐	☐	☐	☐	☐	☐	☐
C3.3 L'innovation considère des questions de sexe et de genre*.	☐	☐	☐	☐	☐	☐	☐
C3.4 L'innovation respecte les communautés autochtones et les minorités visibles* et leur[s] culture[s].	☐	☐	☐	☐	☐	☐	☐
C3.5 La mise à l'échelle de l'innovation suit des lignes directrices visant à garantir l'équité, la diversité et l'inclusion des groupes vulnérables* dans la société.	☐	☐	☐	☐	☐	☐	☐

* La définition des termes employés est présentée dans le glossaire.

Notes ✎

5.4 C4 : Contexte politique de la mise à l'échelle

Cette composante rassemble des énoncés liés aux barrières existantes dans le contexte politique de la mise à l'échelle. Ce sont des obstacles politiques puisqu'ils se situent au niveau de la prise de décision politique et de la mise en œuvre des politiques publiques. Ils peuvent être de différentes natures, telles que les conflits d'intérêts entre différents groupes, la bureaucratie, la résistance institutionnelle au changement et les contraintes légales et réglementaires. De plus, ces obstacles peuvent se situer à différents niveaux, du local au national et, dans certains cas, même au niveau international. Ils peuvent résulter de facteurs structurels ou systémiques qui rendent difficile la mise à l'échelle de l'innovation. La mise à l'échelle doit s'aligner aux programmes et aux plans des autorités à différents niveaux, tels que l'OMS, les ministères de la santé des pays et d'autres organismes de réglementation locaux, nationaux et internationaux.

Veuillez répondre à chacun des énoncés suivants en indiquant le chiffre qui décrit le mieux votre opinion :

	Pas du tout d'accord 1	2	3	4	5	6	Tout à fait d'accord 7
C4.1 Il n'y a pas d'obstacle politique* à la mise à l'échelle de cette innovation.	☐	☐	☐	☐	☐	☐	☐
C4.2 La mise à l'échelle de l'innovation est alignée à la législation nationale et locale.	☐	☐	☐	☐	☐	☐	☐

* La définition des termes employés est présentée dans le glossaire.

Notes ✎

5.5 C5 : Efficacité de l'innovation mise à l'échelle

Cette composante rassemble des énoncés liés aux données probantes sur l'efficacité de l'innovation mise à l'échelle. Il s'agit des données pertinentes et de qualité en tenant compte des impacts de l'innovation à court, moyen et long terme. Ces impacts peuvent être positifs, comme l'amélioration de la santé et du bien-être de la population et des services, ou négatifs, comme les risques ou les événements adverses.

Veuillez répondre à chacun des énoncés suivants en indiquant le chiffre qui décrit le mieux votre opinion :

	Pas du tout d'accord 1	2	3	4	5	6	Tout à fait d'accord 7
C5.1 Il existe des données pertinentes et de qualité* sur l'efficacité* de l'innovation.	☐	☐	☐	☐	☐	☐	☐
C5.2 Les avantages de l'innovation et les impacts positifs sur la santé et le bien-être des individus et des communautés sont facilement démontrés par des données probantes.	☐	☐	☐	☐	☐	☐	☐
C5.3 Il existe des données pertinentes et de qualité* sur les désavantages de l'innovation.	☐	☐	☐	☐	☐	☐	☐
C5.4 Les désavantages potentiels **de la non mise à l'échelle** ont été considérés.	☐	☐	☐	☐	☐	☐	☐

* La définition des termes employés est présentée dans le glossaire.

Notes ✎

5.6 C6 : Coûts de la mise à l'échelle

Cette composante rassemble des énoncés liés aux coûts de la mise à l'échelle de l'innovation. Il est important de s'assurer que les coûts totaux nécessaires à la mise à l'échelle peuvent être financés (ressources matérielles, humaines, financières etc.). En plus, ces coûts doivent être justifiables par rapport aux bénéfices quantifiables de la mise à l'échelle de l'innovation (coût-efficacité). Également, il est intéressant de comparer le coût-efficacité de la mise à l'échelle de l'innovation avec le coût-efficacité d'autres alternatives existantes (des autres innovations, des programmes de santé existants etc.) afin de justifier que la mise à l'échelle sera avantageuse.

Veuillez répondre à chacun des énoncés suivants en indiquant le chiffre qui décrit le mieux votre opinion :

	Pas du tout d'accord 1	2	3	4	5	6	Tout à fait d'accord 7
C6.1 Il existe des données pertinentes et de qualité* sur les ressources matérielles, financières et humaines (coûts totaux) nécessaires à la mise à l'échelle de l'innovation.	☐	☐	☐	☐	☐	☐	☐
C6.2 L'innovation nécessite des ressources matérielles, humaines et financières dont on peut minimalement s'attendre à ce qu'elles soient disponibles pendant la mise à l'échelle	☐	☐	☐	☐	☐	☐	☐
C6.3 Il existe des données pertinentes et de qualité* sur le coût-efficacité de la mise à l'échelle de l'innovation (en comparaison aux alternatives existantes).	☐	☐	☐	☐	☐	☐	☐

* La définition des termes employés est présentée dans le glossaire.

Notes ✎

5.7 C7 : Adaptabilité de l'innovation mise à l'échelle

Cette composante rassemble des énoncés liés à l'adaptabilité de l'innovation aux nouveaux contextes. L'adaptation des innovations au cours de la mise à l'échelle est nécessaire en fonction des différents milieux, des valeurs et des besoins des populations ciblées, ainsi que des changements au fil du temps. Parallèlement, la cohérence avec les caractéristiques, les objectifs et, surtout, les impacts positifs de l'innovation doit être maintenue même si les adaptations sont nécessaires lors de la mise à l'échelle.

Veuillez répondre à chacun des énoncés suivants en indiquant le chiffre qui décrit le mieux votre opinion :

	Pas du tout d'accord 1	2	3	4	5	6	Tout à fait d'accord 7
C7.1 Il existe des données pertinentes et de qualité* sur l'adaptabilité de l'innovation.	☐	☐	☐	☐	☐	☐	☐
C7.2 Ce qui est nécessaire pour adapter l'innovation localement (nouveau contexte*) a été considéré.	☐	☐	☐	☐	☐	☐	☐
C7.3 Des adaptations peuvent être (ou ont pu être) apportées à l'innovation sans altérer les caractéristiques, les objectifs et les résultats fondamentaux.	☐	☐	☐	☐	☐	☐	☐

* La définition des termes employés est présentée dans le glossaire.

Notes ✎

5.8 C8 : Couverture de la mise à l’échelle

Cette composante rassemble des énoncés liés au potentiel de couverture et d’adoption de l’innovation après la mise à l’échelle. La couverture fait référence au nombre de personnes ou services qui bénéficient de la mise à l'échelle. L'adoption implique l'intégration de l'innovation dans les pratiques existantes, ce qui peut varier depuis l'intention de l'adopter jusqu'à l'adoption partielle ou totale par des individus ou des services.

Veuillez répondre à chacun des énoncés suivants en indiquant le chiffre qui décrit le mieux votre opinion :

	Pas du tout d'accord 1	2	3	4	5	6	7 Tout à fait d’accord
C8.1 Il y a une définition claire de qui est la population ciblée* par la mise à l’échelle de l’innovation (c’est-à-dire qui sera couvert et quels sont leurs attributs).	☐	☐	☐	☐	☐	☐	☐
C8.2 La mise à l’échelle de l'innovation a le potentiel de couvrir l'ensemble des populations ciblées*.	☐	☐	☐	☐	☐	☐	☐
C8.3 Il existe des données pertinentes et de qualité sur l'adoption de l'innovation en considérant le nombre d'unités (personnes, services, etc.) prévues pour adopter l'innovation et le nombre réel qui l'adopte.	☐	☐	☐	☐	☐	☐	☐

* La définition des termes employés est présentée dans le glossaire.

Notes ✎

5.9 C9 : Acceptabilité de l'innovation mise à l’échelle

Cette composante rassemble des énoncés liés à l’acceptabilité de l’innovation mise à l’échelle. Elle devra être bien acceptée et considérée comme appropriée parmi les parties prenantes et la population ciblée par la mise à l’échelle.

Veuillez répondre à chacun des énoncés suivants en indiquant le chiffre qui décrit le mieux votre opinion :

	Pas du tout d'accord 1	2	3	4	5	6	Tout à fait d’accord 7
C9.1 Il existe des données pertinentes et de qualité sur l'acceptabilité de l'innovation parmi les populations ciblées*.	☐	☐	☐	☐	☐	☐	☐
C9.2 L’innovation est présentée de façon appropriée en utilisant un langage accessible* pour les populations ciblées*.	☐	☐	☐	☐	☐	☐	☐

* La définition des termes employés est présentée dans le glossaire.

Notes ✎

5.10 C10 : Milieu de mise à l'échelle

Cette composante rassemble des énoncés liés au milieu où l'innovation sera mise à l'échelle. Il est important que ce contexte soit suffisamment similaire ou compatible avec le contexte pilote ou expérimental à partir duquel les données probantes sur l'efficacité de l'innovation ont été recueillies. En outre, il est nécessaire que les principaux partenariats soient présents localement pour la mise à l'échelle.

Veuillez répondre à chacun des énoncés suivants en indiquant le chiffre qui décrit le mieux votre opinion :

	Pas du tout d'accord 1	2	3	4	5	6	Tout à fait d'accord 7
C10.1 L'innovation a été testée dans le même type de milieu où elle sera mise à l'échelle.	☐	☐	☐	☐	☐	☐	☐
C10.2 Les partenariats multipartites locaux* sont en place pour soutenir la mise à l'échelle.	☐	☐	☐	☐	☐	☐	☐
C10.3 Il existe des ressources humaines qualifiées* disponibles dans le milieu où l'innovation sera mise à l'échelle.	☐	☐	☐	☐	☐	☐	☐

* La définition des termes employés est présentée dans le glossaire.

Notes ✎

5.11 C 11 : Infrastructure requise pour la mise à l'échelle

Cette composante rassemble des énoncés liés à l'infrastructure requise pour la mise à l'échelle de l'innovation. La faisabilité exige que les différents types d'infrastructures nécessaires soient réalisables et en place lors de la mise à l'échelle. Une infrastructure organisationnelle implique d'obtenir les autorisations institutionnelles nécessaires, de mettre en place un comité de gouvernance et d'obtenir un soutien politique et communautaire pour la mise à l'échelle. Une infrastructure de suivi et d'évaluation signifie que du personnel qualifié, des mesures et des équipements seront disponibles tout au long du processus pour surveiller et évaluer la mise à l'échelle de l'innovation.

Veuillez répondre à chacun des énoncés suivants en indiquant le chiffre qui décrit le mieux votre opinion :

	Pas du tout d'accord 1	2	3	4	5	6	Tout à fait d'accord 7
C11.1 Il existe des données pertinentes et de qualité* sur la faisabilité de la mise à l'échelle de l'innovation.	☐	☐	☐	☐	☐	☐	☐
C11.2 Les exigences d'infrastructure pour la mise à l'échelle de l'innovation sont réalisables.	☐	☐	☐	☐	☐	☐	☐
C11.3 L'infrastructure organisationnelle* requise est disponible pour la mise à l'échelle de l'innovation.	☐	☐	☐	☐	☐	☐	☐
C11.4 Des structures sont en place pour **surveiller** le processus de mise à l'échelle.	☐	☐	☐	☐	☐	☐	☐
C11.5 Des structures sont en place pour **évaluer** le processus de mise à l'échelle.	☐	☐	☐	☐	☐	☐	☐

Notes ✎

5.12 C12 : Pérennisation

Cette composante rassemble des énoncés liés à la pérennisation de l'innovation. Cela implique de réfléchir à la disponibilité des ressources matérielles, humaines et financières dans le temps. La pérennisation de la mise à l'échelle se réfère à la capacité de maintenir et de soutenir la mise à l'échelle de l'innovation d'une façon durable et continue.

Veuillez répondre à chacun des énoncés suivants en indiquant le chiffre qui décrit le mieux votre opinion :

	Pas du tout d'accord 1	2	3	4	5	6	Tout à fait d'accord 7
C12.1 La pérennisation de la mise à l'échelle a été considérée.	☐	☐	☐	☐	☐	☐	☐
C12.2 Les ressources matérielles, humaines et financières requises pour la mise à l'échelle de l'innovation sont pérennes.	☐	☐	☐	☐	☐	☐	☐

* La définition des termes employés est présentée dans le glossaire.

Notes ✎

6. Ressources utilisées pour construire l'outil

Vous trouverez ici la liste des outils dont les éléments ont été sélectionnés pour composer l'outil ISSaQ 4.0 :

Baker PRA, Shipp JJ, Wellings SH, Priest N, Francis DP. Assessment of applicability and transferability of evidence-based antenatal interventions to the Australian indigenous setting. Health Promot Int. 2012;27:208–19.

Ben Charif A, Hassani K, Wong ST, Zomahoun HTV, Fortin M, Freitas A, et al. Assessment of scalability of evidence-based innovations in community-based primary health care: a cross-sectional study. CMAJ Open. 2018;6:E520–7.

Ben Charif A, Zomahoun HTV, Massougbodji J, Khadhraoui L, Pilon MD, Boulanger E, et al. Assessing the scalability of innovations in primary care: a cross-sectional study. CMAJ Open. 2020;8:E613–8."

Bennett S, Mahmood SS, Edward A, Tetui M, Ekirapa-Kiracho E. Strengthening scaling up through learning from implementation: comparing experiences from Afghanistan, Bangladesh and Uganda. Health Res Policy Syst. 2017;15:108.

Bhattacharyya O, Wu D, Mossman K, Hayden L, Gill P, Cheng Y-L, et al. Criteria to assess Potential reverse innovations: opportunities for shared learning between high- and low-income countries. Glob Health. 2017;13:4."

Buffett C, Ciliska D, Thomas H. Can I Use This Evidence in my Program Decision? Assessing Applicability and Transferability of Evidence [Internet]. Hamilton, ON, Canada; 2007 [cited 2020 Aug 21]. Available from: https://www.nccmt.ca/uploads/media/media/0001/01/110008a2754f35048bb7e8ff446117133b81ab13.pdf"

Buffett C, Ciliska D, Thomas H. Évaluation de l'applicabilité et de la transférabilité des données probantes : Puis-je utiliser ces données probantes dans mes décisions de programmes? [Internet]. Hamilton, ON, Canada; 2007 [cited 2020 Aug 21]. Available from: https://www.nccmt.ca/uploads/media/media/0001/01/ea0f35a0458f84bce52deabc21c4a57ff6a818f6.pdf

Burchett H, Umoquit M, Dobrow M. How do we know when research from one setting can be useful in another? A review of external validity, applicability and transferability frameworks. J Health Serv Res Policy. 2011;16:238–44.

Burchett HED, Mayhew SH, Lavis JN, Dobrow MJ. When can research from one setting be useful in another? Understanding perceptions of the applicability and transferability of research. Health Promot Int. 2013;28:418–30.

Cambon L, Minary L, Ridde V, Alla F. A tool to analyze the transferability of health promotion interventions. BMC Public Health. 2013;13:1184."

Cambon L, Minary L, Ridde V, Alla F. Transferability of interventions in health education: a review. BMC Public Health. 2012;12:497.

Cambon L, Minary L, Ridde V, Alla F. Un outil pour accompagner la transférabilité des interventions en promotion de la santé : ASTAIRE. Sante Publique (Bucur). S.F.S.P.; 2014;Vol. 26:783–6.

Milat AJ, King L, Bauman AE, Redman S. The concept of scalability: increasing the scale and potential adoption of health promotion interventions into policy and practice. Health Promot Int. 2013;28:285–98.

Milat AJ, Lee K, Conte K, Grunseit A, Wolfenden L, van Nassau F, et al. Intervention Scalability Assessment Tool: A decision support tool for health policy makers and implementers. Health Res Policy Syst. 2020;18:1.

Milat AJ, Newson R, King L, Rissel C, Wolfenden L, Bauman A, et al. A guide to scaling up population health interventions. Public Health Res Pract. 2016;26:e2611604.

Milat AJ, Newson R, King L. Increasing the Scale of Population Health Interventions: A Guide [Internet]. Evidence and Evaluation Guidance Series, Population and Public Health Division. Sydney: NSW Ministry of Health: Centre for Epidemiology and Evidence; 2014 [cited 2020 Aug 19]. Available from: https://www.health.nsw.gov.au/research/Pages/scalability-guide.aspx"

Morinière LC, Turnbull M, Bremaud I, Vaughan-Lee H, Xaxa V, Farheen SA. Toolkit: Scalability Assessment and Planning (SAP) (including workshop guidance) [Internet]. 2018 [cited 2020 Aug 21]. Available from: https://resourcecentre.savethechildren.net/library/scalability-assessment-and-planning-sap-toolkit

Organisation mondiale de la santé (OMS). Avoir le but à l'esprit dès le début : la planification des projets pilotes et d'autres recherches programmatiques pour un passage à grande échelle réussi [Internet]. World Health Organization, Department of Reproductive Health and Research - ExpandNet; 2013 [cited 2020 Aug 19]. Available from: https://www.who.int/reproductivehealth/publications/strategic_approach/9789241502320/fr/"

Organisation mondiale de la santé (OMS). Neuf étapes pour élaborer une stratégie de passage à grande échelle [Internet]. 2011 [cited 2020 Aug 19]. Available from: https://www.who.int/reproductivehealth/publications/strategic_approach/9789241500319/fr/

Organización mundial de la salud (OMS). Nueve pasos para formular una estrategia de ampliación a escala [Internet]. World Health Organization; 2011 [cited 2020 Aug 19]. Available from: https://www.who.int/reproductivehealth/publications/strategic_approach/9789241500319/es/

Spicer N, Bhattacharya D, Dimka R, Fanta F, Mangham-Jefferies L, Schellenberg J, et al. "Scaling-up is a craft not a science": Catalysing scale-up of health innovations in Ethiopia, India and Nigeria. Soc Sci Med 1982. 2014;121:30–8.

Vaughan-Lee H, Moriniere LC, Bremaud I, Turnbull M. Understanding and measuring scalability in disaster risk reduction. Disaster Prev Manag Int J. Emerald Publishing Limited; 2018;27:407–20.

Wang S, Moss JR, Hiller JE. Applicability and transferability of interventions in evidence-based public health. Health Promot Int. 2006;21:76–83.

World Health Organization (WHO). Beginning with the end in mind: planning pilot projects and other programmatic research for successful scaling up [Internet]. World Health Organization; 2018 [cited 2020 Aug 19]. Available from: https://www.who.int/reproductivehealth/publications/strategic_approach/9789241502320/en/

World Health Organization (WHO). Nine steps for developing a scaling-up strategy [Internet]. 2010 [cited 2018 Apr 26]. Available from: http://www.who.int/reproductivehealth/publications/strategic_approach/9789241500319

---

[i] World Health Organization. Nine Steps for Developing a Scaling-Up Strategy. Geneva: WHO ExpandNet; 2010

[ii] World Health Organization. Nine Steps for Developing a Scaling-Up Strategy. Geneva: WHO ExpandNet; 2010

[iii] Sheridan DJ. Research: increasing value, reducing waste. The Lancet. 2014;383:1123. ; Fixsen D, Blase K, Metz A, Van Dyke M. Statewide Implementation of Evidence-Based Programs. Exceptional Children. 2013;79:213–30. ; Bégin M, Eggertson L, Macdonald N. A country of perpetual pilot projects. CMAJ. 2009;180:1185, E88-89.

[iv] World Health Organization. Nine Steps for Developing a Scaling-Up Strategy. Geneva: WHO ExpandNet; 2010. ; Ben Charif A, Hassani K, Wong ST, Zomahoun HTV, Fortin M, Freitas A, et al. Assessment of scalability of evidence-based innovations in community-based primary health care: a cross sectional study. CMAJ Open. 2018;6:E520–7. ; Ben Charif A, Zomahoun HTV, Massougbodji J, Khadhraoui L, Pilon MD, Boulanger E, et al. Assessing the scalability of innovations in primary care: a cross-sectional study. CMAJ Open. 2020;8:E613–8. ; Cooley L, Linn JF. Taking innovations to scale: Methods, applications and lessons: Washington DC: Management Systems International; 2014. ; Centre for Epidemiology and Evidence, et al. Increasing the Scale of Population Health Interventions: A Guide. In: Evidence and Evaluation Guidance Series. Sydney: NSW Ministry of Health; 2014.

[v] Milat AJ, et al. The concept of scalability: increasing the scale and potential adoption of health promotion interventions into policy and practice. Health Promot Int. 2013;28(3):285–98.

[vi] Ben Charif A, Zomahoun HTV, Gogovor A, Abdoulaye Samri M, Massougbodji J, Wolfenden L, Ploeg J, Zwarenstein M, Milat AJ, Rheault N, Ousseine YM, Salerno J, Markle-Reid M, Légaré F. Tools for assessing the scalability of innovations in health: a systematic review. Health Res Policy Syst. 2022 Mar 24;20(1):34. doi: 10.1186/s12961-022-00830-5. PMID: 35331260; PMCID: PMC8943495. ; Milat, A., Lee, K., Conte, K. et al. Intervention Scalability Assessment Tool: A decision support tool for health policy makers and implementers. Health Res Policy Sys 18, 1 (2020). https://doi.org/10.1186/s12961-019-0494-2

[vii] Ben Charif A, Hassani K, Wong ST, Zomahoun HTV, Fortin M, Freitas A, et al. Assessment of scalability of evidence-based innovations in community-based primary health care: a cross-sectional study. CMAJ Open. 2018;6:E520–7. ; Ben Charif A, Zomahoun HTV, Massougbodji J, Khadhraoui L, Pilon MD, Boulanger E, et al. Assessing the scalability of innovations in primary care: a cross-sectional study. CMAJ Open. 2020;8:E613–8.

[viii] Ben Charif, A., Zomahoun, H., LeBlanc, A., Langlois, L., Wolfenden, L., Yoong, S. L., Williams, C. M., Lépine, R., & Légaré, F. (2017). Effective strategies for scaling up evidence-based practices in primary care: a systematic review. Implementation science : IS, 12(1), 139. https://doi.org/10.1186/s13012-017-0672-y

[ix] Milat AJ, Newson R, King L, et al. A guide to scaling up population health interventions. Public Health Res Pract 2016;26:e2611604. ; Nine steps for developing a scaling-up strategy. Geneva: World Health Organization; 2010. Available: www.who.int/reproductivehealth/publications/strategic_approach/9789241500319/en/ (accessed 2018 Apr. 26).

[x] Ben Charif A, Zomahoun HTV, Gogovor A, Abdoulaye Samri M, Massougbodji J, Wolfenden L, Ploeg J, Zwarenstein M, Milat AJ, Rheault N, Ousseine YM, Salerno J, Markle-Reid M, Légaré F. Tools for assessing the scalability of innovations in health: a systematic review. Health Res Policy Syst. 2022 Mar 24;20(1):34. doi: 10.1186/s12961-022-00830-5. PMID: 35331260; PMCID: PMC8943495.

[xi] Peasgood, T., Mukuria, C., Carlton, J., Connell, J., & Brazier, J. (2020, 2020/12/07). Criteria for item selection for a preference-based measure for use in economic evaluation. Quality of Life Research. https://doi.org/10.1007/s11136-020-02718-9.
