# Supplementary material for: Engagement of Older Adults Receiving Home Care Services and Their Caregivers in Health Decisions in Partnership With Clinical Teams: Protocol for a Multimethod Study to Prioritize and Culturally Adapt Decision Aids for Home Care
Source: JMIR Res Protoc. 2023 Nov 20;12:e53150. doi: 10.2196/53150 (PMC10696497; doi:10.2196/53150)
Supplement: Multimedia Appendix 2 [file resprot_v12i1e53150_app2.pdf]

**Review Type/Type d'évaluation:**	Committee Member 1/Membre de comité 1
**Name of Applicant/Nom du chercheur:**	Légaré, France
**Application No./Numéro de demande:**	475385
**Agency/Agence:**	CIHR/IRSC
**Competition/Concours:**	2021-10-26 Catalyst Grant: Quadruple Aim and Equity/Subvention Catalyseur : Quatre objectifs et équité
**Committee/Comité:**	Catalyst Grant : Quadruple Aim and Equity/Subvention catalyseur : Quatre objectifs et équité
**Title/Titre:**	ENGAGEment des ainés.es recevant des soins à domicile et de leurs proches dans les décisions en partenariat avec les équipes cliniques

Assessment/Évaluation:

Résumé. L'application provient d'un groupe de 6 chercheurs et 5 d'utilisateurs de connaissances (3 institutionnels et 2 patients partenaires) piloté par D^{re} Légaré de l'Université Laval. Le projet proposé est un projet de mobilisation des connaissances dans le domaine des soins à domicile (SAD) des aînés et la prise de décision partagée (PDP) comme moyen d'améliorer l'autonomisation des aînés et de leurs proches et de contribuer à leur santé et bien-être. Il porte spécifiquement sur les outils d'aide à la décision (OADs) dans le processus de décision partagée entre les aînés et de leurs proches et les équipes cliniques face aux prestations de soins à domicile. Les objectifs spécifiques du projet sont : 1) d'identifier et de prioriser parmi les outils d'aide à la décision existants, ceux qui ont une capacité de mise à l'échelle et qui répondent aux besoins des utilisateurs de connaissances impliqués du milieu (aînés et les équipes cliniques) ; 2) d'adapter les OADs priorisés à la culture du Québec et de les mettre en format numérique et imprimable ; 3) de les intégrer dans une formation aux équipes cliniques. Pour ce faire, on propose sur 12 mois un processus itératif (revue systématique sur OADs existants spécifiques aux SAD pour établir leur potentiel d'être mis à l'échelle, étude Delphi en ligne (2 tours) avec 60 participants (aînés et proches, intervenants, décideurs et experts en OADs) avec méthode de priorisation RAND pour établir 3 OADs jugés les plus prioritaires, adaptation culturelle des 3 OADs priorisés, conception des 3 OADs en format imprimables et interactifs, formation IP PDP adaptée pour plusieurs OADs en SAD) le tout appuyé par un comité de pilotage (représentant.es des UC, d'aînés et proches, chercheurs et étudiants, stagiaires). L'approche comparative en sexe et genre (ACSG) et d'autres dimensions (PROGRESS+) sera appliquée à toutes les étapes du projet, notamment lors de la conception et la mise en pratique des OADs afin de tenir compte des impacts des différences de sexe, genres, races, statuts socio-économiques et de situation de handicap sur les capacités transversales d'autonomisation des gens.

Mérite scientifique (qualité de l'idée, importance de l'idée, faisabilité de l'approche). Forces : Problématique et rationnel d'utiliser OADs dans SAD clairs et convaincants. Objectifs et démarche proposés permettront de mobiliser les connaissances nécessaires pour établir et mettre en œuvre des OADs adaptés pour aînés, proches aidants et équipes cliniques dans un contexte de prise de décision partagée (PDP) face aux prestations de SAD. Très bonne faisabilité de l'approche pour objectifs #1, #2, #3 avec échéanciers ambitieux mais acceptables. Travaux proposés appuyés par une équipe engagée avec des travaux antérieurs et expertises établies et supportées par un fort engagement des UC. Considérations appropriées liées au sexe et/ou au genre et à l'équité, diversité et inclusion (EDI) Faiblesses : Opérationnalisation (cadre théorique et clinique) de l'utilisation des OADs en contexte de SAD peu développée et non spécifique dans la demande (gagnerait à être développé). L'opérationnalisation de l'objectifs #4 (qui représente 6 mois de travail sur les 12 mois du projet) n'est pas assez développée, est très théorique et générique (cf. utilisation cadre de Chennel pour adaptation culturelle et méthodes de conception centrées sur l'utilisateur d'OADs spécifiques au SAD pour l'adaptation culturelle des OADs) avec des livrables incertains sur ce qui sera produit (formation IP PDP adaptée pour plusieurs OADs) et la maturité attendue (cf. preuve de concept OADs).

Impact potentiel (besoin et importance des résultats escomptés, renforcement du partenariat et des capacités, plan d'application des connaissances). Forces : Excellente adéquation entre thème du projet (PDP) et objectifs de l'appel à projet (quadruple objectif). Objectifs spécifiques du projet développés selon besoins des UC avec mise en

Review Type/Type d'évaluation: Committee Member 1/Membre de comité 1
Name of Applicant/Nom du chercheur: Légaré, France
Application No./Numéro de demande: 475385
Agency/Agence: CIHR/IRSC
Competition/Concours: 2021-10-26 Catalyst Grant: Quadruple Aim and Equity/Subvention Catalyseur : Quatre objectifs et équité
Committee/Comité: Catalyst Grant : Quadruple Aim and Equity/Subvention catalyseur : Quatre objectifs et équité
Title/Titre: ENGAGEment des ainés.es recevant des soins à domicile et de leurs proches dans les décisions en partenariat avec les équipes cliniques

---

Assessment/Évaluation:

œuvre en co-construction avec eux. Engagement et partenariat avec les UC depuis 2007. Excellent potentiel de consolidation et expansion du partenariat entre équipe de recherche et UC sur OADS appliqués en SAD avec des bonnes perspectives pour renforcer les capacités de recherche (cf. financements complémentaires pour application des OADs en SAD). Faiblesses : Résultats escomptés auront des impacts intéressants pour la promotion des OADs en contexte SAD mais seront spécifiques au milieu d'UC visé (généralisation ?). Plan d'application des connaissances acceptable mais un peu générique.

**Review Type/Type d'évaluation:**	Committee Member 2/Membre de comité 2
**Name of Applicant/Nom du chercheur:**	Légaré, France
**Application No./Numéro de demande:**	475385
**Agency/Agence:**	CIHR/IRSC
**Competition/Concours:**	2021-10-26 Catalyst Grant: Quadruple Aim and Equity/Subvention Catalyseur : Quatre objectifs et équité
**Committee/Comité:**	Catalyst Grant : Quadruple Aim and Equity/Subvention catalyseur : Quatre objectifs et équité
**Title/Titre:**	ENGAGEment des ainés.es recevant des soins à domicile et de leurs proches dans les décisions en partenariat avec les équipes cliniques

Assessment/Évaluation:

Légaré, France

ÉVALUATION # 475385

COTE DE LA DEMANDE

Résumé de la demande :

Le projet s’intéresse à la question de la prise de décision partagée (PDP) pour mieux répondre aux besoins des ainé.es et leurs proches confronté.es à des décisions en santé et services sociaux, et ce, en étudiant plus spécifiquement les outils d’aide à la décision (OADs) comme des interventions ciblant les patient.es et favorisant la PDP dans le domaine des soins et services à domicile.

Les **objectifs spécifiques** sont: **1)** Établir un partenariat étroit avec l’ensemble des utilisateurs de connaissances (UC); **2)** Évaluer la capacité de mise à l’échelle des OADs identifiés lors d’une revue systématique complétée par notre équipe; **3)** Prioriser les OADs qui répondent aux besoins des UC; **4)** Jeter les bases d’une adaptation des OADs priorisés au contexte des UC qui pourront être intégrés à une formation aux équipes cliniques en SAD; et **5)** Renforcer les capacités dans le partenariat et la recherche en SAD et soins aux ainé.es.

Misant sur des méthodes mixtes et participatives, sur un partenariat de longue date et sur une production scientifique riche dans ce domaine, l’équipe entend jeter les bases d’une implantation à large échelle d’ OADs spécifiques aux SAD et culturellement adaptés pour le Québecet renforcer l’autonomisation ainsi que la santé et le bien-être des ainé.es qui reçoivent des SAD et leurs proches.

A. MÉRITE SCIENTIFIQUE

1. CONCEPT

• Qualité de l'idée

Le projet comporte des objectifs clairs et bien définis, et repose sur des travaux extensifs de recherche et de synthèse de connaissance menés par cette équipe qui a une expertise de classe mondiale dans le champ de la décision partagée et des outils d’aides à la décision. L’originalité du projet réside dans le fait que cette approche soit déployée dans un champ moins exploré, celui de soins à domicile. L’acceptabilité et l’intérêt stratégique du projet ont été validés auprès d’un groupe diversifié de parties prenantes du domaine.

Il apparaît tout à fait plausible que le projet proposé puisse générer des résultats pertinents qui contribuent à

Review Type/Type d'évaluation: Committee Member 2/Membre de comité 2
Name of Applicant/Nom du chercheur: Légaré, France
Application No./Numéro de demande: 475385
Agency/Agence: CIHR/IRSC
Competition/Concours: 2021-10-26 Catalyst Grant: Quadruple Aim and Equity/Subvention Catalyseur : Quatre objectifs et équité
Committee/Comité: Catalyst Grant : Quadruple Aim and Equity/Subvention catalyseur : Quatre objectifs et équité
Title/Titre: ENGAGEment des ainés.es recevant des soins à domicile et de leurs proches dans les décisions en partenariat avec les équipes cliniques

---

Assessment/Évaluation:

l'amélioration des connaissances dans un champ d'application important en lien avec l'ensemble des quatre objectifs et de l'équité en santé pour tous.

• Importance de l'idée

Les auteurs documentent bien que la PDP améliore les expériences de soins, les issues de santé, le bien-être des équipes cliniques et l'efficience (i.e. quatre objectifs). Ils apportent des preuves à l'effet que les ainé.es ayant des besoins de SAD sont très peu engagé.es dans les décisions concernant leur santé. Les auteurs présentent des arguments convaincants à l'effet que ces travaux seront en mesure de produire des résultats probants originaux qui contribueront à répondre efficacement des besoins non-comblés.

2. FAISABILITÉ

• Approche

L'approche proposée repose sur des cadres conceptuels éprouvés. Les méthodes sont simples mais appropriées et applicables dans le cadre d'un projet d'un an et compatibles avec le niveau de financement demandé.

Les méthodes et l'intégration du sexe et du genre sont clairement explicitées. L'approche comparative en sexe et genre (ACSG) et d'autres dimensions (PROGRESS+) sont décrites aux différentes étapes de réalisation.

Les auteurs fournissent l'historique détaillé de leurs nombreuses réalisations de recherche en lien avec les utilisateurs des connaissances dans ce domaine dans la région de la Capitale nationale, de l'est du Québec, au niveau provincial et au niveau pancanadien. Il ne fait pas de doute que l'équipe détient les acquis et l' expertise pour assurer la participation significative des UC tout au long du processus de recherche.

Les délais d'exécution associés aux livrables du projet apparaissent réalistes.

La proposition fait mention de difficultés potentielles et de stratégies d'atténuation appropriées en lien avec les difficultés de recrutement, la priorisation et la pandémie.

3. EXPERTISE ET EXPÉRIENCE DE L'ÉQUIPE

L'équipe interdisciplinaire candidate est très solide et collabore de longue date. Elle possède une expérience et expertise de fort calibre. Le niveau d'engagement ou de participation de l'équipe candidate ne fait pas de doute. Le contexte apparaît facilitant.

**Review Type/Type d'évaluation:**	Committee Member 2/Membre de comité 2
**Name of Applicant/Nom du chercheur:**	Légaré, France
**Application No./Numéro de demande:**	475385
**Agency/Agence:**	CIHR/IRSC
**Competition/Concours:**	2021-10-26 Catalyst Grant: Quadruple Aim and Equity/Subvention Catalyseur : Quatre objectifs et équité
**Committee/Comité:**	Catalyst Grant : Quadruple Aim and Equity/Subvention catalyseur : Quatre objectifs et équité
**Title/Titre:**	ENGAGEment des ainés.es recevant des soins à domicile et de leurs proches dans les décisions en partenariat avec les équipes cliniques

Assessment/Évaluation:

B. IMPACT DE LA RECHERCHE

• La pandémie ainsi que les orientations fédérales et provinciales courantes confirment le caractère prioritaire du projet. Il apparaît hautement probable que le projet offre le potentiel d’éclairer les décisions à venir en lien avec le renforcement et le renouvellement des soins à domicile au pays, dans la perspective des quatre objectifs et de l'équité en santé pour tous.

Le plan d'ACi de l'équipe mise sur les partenariats déjà établis. Le plan de mobilisation des connaissances en fin de subvention est peu élaboré, ce qui est probablement le seul point plus faible de cette demande de très grande qualité.

BUDGET DEMANDÉ ET RECOMMANDATION OFFICIELLE

Le budget semble justifié. L’intégration des considérations relatives au sexe et/ou au genre dans la proposition est une force. Pas d’enjeux éthiques non discuté dans la proposition.

**Review Type/Type d'évaluation:**	SO Notes /Notes de l'agent scientifique
**Name of Applicant/Nom du chercheur:**	Légaré, France
**Application No./Numéro de demande:**	475385
**Agency/Agence:**	CIHR/IRSC
**Competition/Concours:**	2021-10-26 Catalyst Grant: Quadruple Aim and Equity/Subvention Catalyseur : Quatre objectifs et équité
**Committee/Comité:**	Catalyst Grant : Quadruple Aim and Equity/Subvention catalyseur : Quatre objectifs et équité
**Title/Titre:**	ENGAGEment des ainés.es recevant des soins à domicile et de leurs proches dans les décisions en partenariat avec les équipes cliniques

Assessment/Évaluation:

Based on the initial assessment of the assigned reviewers, your application was considered to be highly competitive and recommended for funding without further discussion. As a result, no Scientific Officer notes were generated. Your reviews are available on ResearchNet.

****

D’après l’évaluation initiale des évaluateurs désignés, votre demande a été jugée hautement compétitive et a été recommandée pour un financement sans autre discussion. Par conséquent, l’agent scientifique n’a produit aucune note. Vos évaluations sont accessibles dans RechercheNet.
